# Correspondence

**Published:** 1901-02

**Authors:** Charles P. Lyman

**Affiliations:** Dean, School of Veterinary Medicine in Harvard University; Boston


### CORRESPONDENCE.

Editor Journal of Comparative Medicine :

Sir : I have read with great interest the communication on "Veterinary Education" which appeared in the December issue of your valuable periodical, and beg to thank your correspondent, "Yankee," for his kindly expressions in regard to the Harvard Veterinary School and to congratulate him upon his thoughtful and most valuable contribution to a subject which I feel sure is considered to be one of vital importance not only to those of us who are connected with the schools, but to all members of the profession who desire to see the science of animal medicine take its proper rank among the learned professions. If veterinarians generally would discuss the question in the same painstaking manner and with the broadness of view of your correspondent, through the journals, it would serve to clear the minds of the members, unify the profession upon some certain policy, and aid and strengthen the schools. There are one or two points in the letter under consideration upon which I shall like, with your permission, to make some remarks, not, however, in any spirit of contention, but that a fuller and more correct appreciation may be had of the work so far done at the Harvard School.

Your correspondent writes : "The original mistake that was made appears to have been based on the supposition that a veterinary school required no equipment in addition to that of a good medical school excepting a hospital, a farriery, and a dissecting-room," etc.

To the full and fair consideration of this criticism, as well as to some of those which follow, it must be thoroughly understood that what was actually done by the management cannot be taken as an evidence of what it would have done had there been any money whatever at its disposition other than that which it was able to earn by its own exertions. The buildings, such as they are—and, on the whole, they have answered *their* purpose admirably—were put up and the land upon which they stand was purchased by men who were willing to put their capital into the desired buildings, with the understanding that they should receive 6 per cent. interest and taxes upon the entire amount expended by them ; they also demanded, further, that the payment of these annual charges should be satisfactorily guaranteed to them for five years.

This guarantee was furnished, but not by Harvard University. The management was, therefore, forced to consider measures which would surely lead to the earning of this sum. The hospital and farriery were built, and all of the obligations to the owners of the building have been discharged, for the last eighteen years, from the earnings of those two divisions of the school, which have also been able to contribute a varying but material sum each year toward offsetting the deficit, coming to the general account from the actual cost of teaching students, which has always been considerably in excess of income derived from that source. The class-rooms used at first were those which are at the Bussey Institute, which is some half-hour's ride by train from Boston. This arrangement was found to be impracticable, because so much time was lost in going and coming between the two places; and it was thereupon determined to put up a new building, to contain the necessary class-rooms, in connection with the hospital. The funds to do this were supplied by the University, but its entire cost was charged to the veterinary school as so much cash advanced, and the school was again obliged to pay 6 per cent. interest upon this added amount from its own earnings.

The connection with the medical school arose, in the first place, through this same necessity for economy; well-fitted laboratories and good teaching had to be provided in such subjects as chemistry, physiology, histology, etc. The medical school generously offered these, and veterinary students were, during our earlier years, taught these fundamental subjects in classes with medical students. Although it was fully realized at the time that, as pointed out by your correspondent, veterinary students should have special instruction in most of these subjects, it was still felt that the desired end could be reached more quickly and surely by beginning with good material at hand rather than by instituting, at that time, independent but inferior courses for veterinary students. Not only were the funds not available, but it would have then been almost impossible, as, indeed, it is still extremely difficult, to obtain properly equipped teachers.

The soundness of this opinion seems to the writer to have been fully proved, for now all of the teaching at the school in physiology, physiological and medical chemistry, histology, pathology, pathological histology, and bacteriology is being given, as are all of the other courses, in exercises especially designed for veterinary students and for them alone, in classes by themselves and by men who have especially prepared themselves for the work.

The only connection which we now have with the medical school is that it still, although at much inconvenience because of the sizes of its own classes, gives us the use of its splendidly equipped laboratories at a very nominal fee. The veterinary school always pays and always has paid fair salaries to all of its instructors. It believes that unless this is done the grade of instruction cannot, except in rare individual instances, be maintained at the high grade which is demanded. It also believes in a large teaching staff, because if men are overcrowded or are given a multitude of subjects instruction *cannot* be the best that the individual teacher is capable of giving. This is in accord with the opinion of "Yankee," who says "it has always been necessary for the teachers to divide their time between their classes and their outside pot-boiling work," whereas the school should have had their undivided interest. This is a fact, but that it has been so is but a part of the many difficulties which have been imposed upon the management in its endeavor to carry on a school of high grade without having endowment. It may be said that of late years one of the three veterinarians to whom large parts of the teaching have been intrusted has given his whole time to the school, while the two others have made their "outside practice" almost disastrously subservient to the demands of their work in the school.

It is no more than fair to add here that if the veterinary school had been in possession of its "plant" without the necessity of paying an annual rental upon its entire cost it would to-day show a surplus earning of a few thousands of dollars instead of the deficit which its accounts now show. There is no other department in Harvard University in which an annual rent has to be paid as a part of the cost of its operation.

While it has been the endeavor of the writer to show that the management has been as fully alive as any to the fact that the school has not been all that an ideal veterinary school should be, or as it would have liked to have organized had it been possible, he does not desire to shirk any responsibility which should properly be placed with him, nor does he wish to pretend that many mistakes in judgment have not been made, but he cannot agree with your correspondent as to "the original mistake" in the passage from his letter quoted at the beginning of this paper. The "original mistake" was made in undertaking to organize and maintain the school without *first* having obtained some sort of an endowment, or, as our real estate owners put it, "financial guarantee." But, although this was thought of at the time and some little endeavor to obtain such a fund was made, the prevailing enthusiasm urged the belief that the object was such a good one and its exemplification would be so attractive that the desired funds would soon be forthcoming; and it is even now somewhat of a surprise, as well as a great disappointment, to find that this opinion has not been realized.

Your correspondent, in regretting that Harvard could not have seen that "the day of the veterinary branch of a medical school" was passed when it first began the teaching of veterinary students, goes on to say the experience of the University has shown "that a school of veterinary *medicine* will not succeed even when attached to the best medical school in the country."

The word "attached," as used, is capable of several constructions; but it may be proper to say here that it is now several years since the question of making the veterinary school a branch of the medical school came up in the faculty for very serious consideration. The subject was thoroughly discussed at several meetings, all of which were fully attended, and the unanimous opinion was expressed in the following vote:

"That the teaching of veterinary medicine as a specialty of human medicine is impracticable, because the teaching must be specialized from the very beginning, and, therefore, demands a system of instruction co-ordinate with that of human medicine."

There can be no further doubt as to the position of this school upon that question, nor has it ever been "attached" to the medical school in any way other than that already described.

As to whether or not a mistake has been made in denominating veterinary schools schools of veterinary medicine there is considerable room for further profitable discussion, but the matter hardly comes within the scope of this letter. Would it be any better for us to appear as "attached" to agriculture than to medicine? Why should we be subordinated to any other calling or profession?

I do not think that the remarks of your correspondent upon this division of his communication are by any means as clearly put as the other parts of his letter. There is certainly no argument in saying that "history shows that it is much more successful to combine veterinary science and agriculture." What history does show is that endowed schools are more successful and get along more rapidly than do those not having such aid; and that the schools of Continental Europe, which are maintained at the expense of the several governments, are the most successful of all, whether they are under the Department of War or of Agriculture.

I may repeat what has already been pointed out for the purpose of emphasizing the fact that we now have here special instruction in animal pathology and physiology which embraces *all* of the requirements made by your correspondent, and much more, excepting that we have no herds of cattle, sheep, or swine. It may, however, be said that repeated attempts have been made during the years past to obtain them, all of which have failed simply because of the attending expense. Harvard has the farm and most of the necessary buildings, but this school has not been able to pay for the care and maintenance of the animals.

The feeling, evidently held by your correspondent, that veterinary *science* is not now properly taught in this country is an opinion which is widely held among us, but the exact method by which the desired object is to be reached is a very considerable problem. We all know, as Dr. Huidekoper has quoted, expresses it, that the chief use of the veterinarian " is not that of a tinkerer to treat colic and sew up wounds," and it is exactly this problem that this school has been endeavoring to work out. It began work with the best obtainable material at hand, and made such changes in existing school methods as it thought safe under the conditions which then existed. From that time to this it has continued as fast as it has deemed prudent to institute and insist upon the more scientific development of subjects already being taught and the general careful enlargement of the curriculum. It believes that this is the true way to *develop* the *science* of animal medicine ; it does not believe that, even with unlimited financial means, it would even now be possible to organize a successful school of the highest possible scientific grade. A school to be successful and make its influence felt must have students. Public opinion has not yet been sufficiently educated as to the great possibilities which, some of us see and *know*, are to come through what is now called veterinary medicine, and through that alone. Wonderful progress has been made during the last eighteen years. Just as much is to be and will be done during the next similar period. We must be patient and keep up the work of *gradual development*.

CHARLES P. LYMAN,
Dean, School of Veterinary Medicine in Harvard University.

BOSTON, January 30, 1901.

